# The efficacy and safety of anlotinib combined with platinum-etoposide chemotherapy as first-line treatment for extensive-stage small cell lung cancer: A Chinese multicenter real-world study

**DOI:** 10.3389/fonc.2022.894835

**Published:** 2022-09-20

**Authors:** Hao-Ran Zheng, Ai-Min Jiang, Huan Gao, Na Liu, Xiao-Qiang Zheng, Xiao Fu, Zhi-Ping Ruan, Tao Tian, Xuan Liang, Yu Yao

**Affiliations:** ^1^ Department of Medical Oncology, The First Affiliated Hospital of Xi’an Jiaotong University, Xi’an, China; ^2^ Department of Medical Oncology, Xi’an No.3 Hospital, Xi’an, China

**Keywords:** small cell lung cancer, anlotinib, chemotherapy, real-world data, efficacy, safety

## Abstract

**Background:**

Patients with extensive-stage small-cell lung cancer (ES-SCLC) have high recurrence rates and bleak prognosis. This multicenter real-world study aimed to explore the efficacy and safety of anlotinib combined with platinum-etoposide chemotherapy as the first-line treatment of ES-SCLC.

**Methods:**

Pathologically confirmed ES-SCLC patients receiving anlotinib plus platinum-etoposide chemotherapy as the first-line treatment were enrolled in this retrospective study. The primary endpoint of this study was progression-free survival (PFS), and secondary endpoints included overall survival (OS), objective response rate (ORR), disease control rate (DCR), and adverse reactions. The Cox regression analyses were employed to investigate the independent prognostic factors for OS and PFS of these individuals.

**Results:**

In total, 58 patients were included in this study. The median PFS was 6.0 months [95% confidence interval (CI): 3.5-8.5], and the median OS was 10.5 months (95%CI 8.7-12.3). Thirty-four patients achieved partial response (PR), 18 patients achieved stable disease (SD), and 6 patients achieved progressive disease (PD). The ORR and DCR were 58.6% and 89.6%. The main treatment-related adverse reactions were generally tolerated. Myelosuppression (44.8%) was the most common adverse reaction, followed by hypertension (41.4%), fatigue (34.5%), gastrointestinal reaction (32.7%), and hand-foot syndrome (24.1%). Multivariate analysis showed that post-medication hand-foot syndrome [PFS 8.5 *vs.* 5.5 months, Hazards Ratio (HR)=0.23, 95%CI 0.07-0.72, *P* =0.012] was the independent predictor of PFS, and hypertension (OS 15.9 *vs.* 8.3 months, HR=0.18, 95%CI 0.05-0.58, *P* =0.005) was the independent predictor of OS.

**Conclusion:**

Anlotinib combined with platinum-etoposide chemotherapy as the first-line treatment for ES-SCLC appears to be effective and well-tolerated in the real-world. Well-designed large-scale prospective studies are urgently needed in the future to verify our findings.

## Introduction

Lung cancer is the most frequent cause of tumor death worldwide. Small cell lung cancer (SCLC) is a highly aggressive and deadly malignant tumor, accounting for approximately 10% to 15% of all lung cancers (1–3). SCLC comprises an estimated 250,000 new cases and at least 200,000 deaths worldwide each year (4). Approximately 70% of the patients are diagnosed with extensive-stage SCLC (ES-SCLC) with poor overall survival (OS) (5). It has been reported that the median OS for ES-SCLC patients without systemic therapy is 2 to 4 months (6, 7).

As the gold standard for SCLC therapy, platinum-etoposide chemotherapy has been widely used in the past 40 years. The median progression-free survival (PFS) of platinum-etoposide chemotherapy as the first-line treatment is about 5 months, and the median OS is about 10 months (8). In recent years, the rapid rise of immunotherapy has broken the unshakable position of platinum-etoposide chemotherapy. Atezolizumab, a programmed death-ligand 1 (PD-L1) inhibitor, was studied in IMpower133 clinical trial in combination with platinum-etoposide chemotherapy as the first-line treatment for ES-SCLC. The combined regimen brought survival benefits: the median OS was prolonged for 2 months (12.3 *vs.* 10.3 months), and the 1-year OS rate was increased by 13.5% (51.7% *vs.* 38.2%) compared with platinum-etoposide chemotherapy (9). Durvalumab, another PD-L1 inhibitor, was also found to have a similar OS benefit (13.0 *vs.* 10.3 months) in CASPIAN clinical trial (10). PD-L1 plus platinum-etoposide chemotherapy has become the new first-line therapy for ES-SCLC.

Angiogenesis is a complex process that plays an essential role in tumor growth, invasion and metastasis. Vascular endothelial growth factor (VEGF) is the most critical proangiogenic protein (11). Previous studies found that about 80% of SCLC tissues were positive for VEGF expression, and the VEGF level was an independent prognostic factor in SCLC (12). However, the efficacy of antiangiogenic therapy in SCLC is limited. Bevacizumab, a monoclonal antibody directed against VEGF, showed a promising activity in combination with platinum-etoposide as the first-line treatment of patients with ES-SCLC, and two randomized studies confirmed that bevacizumab improved PFS, but failed to prolong OS (13, 14). Instead, disappointing results have been observed with endostar, sunitinib, sorafenib, vandetanib, and thalidomide in combination with chemotherapy in the first-line setting. Only anlotinib improved PFS and OS as third-line therapy in Chinese patients with SCLC (15). As an oral antiangiogenic tyrosine kinase inhibitor (TKI), anlotinib targets vascular endothelial growth factor receptor (VEGFR), platelet-derived growth factor receptors (PDGFR), fibroblast growth factor receptor (FGFR), and c-kit (16). Based on ALTER 1202 study, anlotinib was approved by the China Food and Drug Administration (CFDA) in 2019 as the third-line and above treatment for SCLC (17). Additionally, some small sample size clinical trials in China have shown the favorable efficacy of anlotinib combined with platinum-etoposide chemotherapy (18–20). The 2021 American society of clinical oncology (ASCO) meeting announced the preliminary result of a phase II clinical study on the efficacy and safety of anlotinib combined with platinum-etoposide chemotherapy in the first-line treatment of ES-SCLC. Twenty patients could evaluate the efficacy, of which the median PFS was 10.0 months, the median OS was 15.0 months, the objective response rate (ORR) was 90%, and the disease control rate (DCR) was 100% (18). It was significantly higher than that of traditional chemotherapy.

In clinical trials, patients are strictly screened. Thus, patients with poor conditions, such as the elderly, combined brain metastases, and the Eastern Cooperative Oncology Group Performance Status (ECOG PS) ≥2, are often excluded. Therefore, we conducted this multicenter retrospective study to investigate the real-world efficacy and safety of anlotinib combined with platinum-etoposide chemotherapy as the first-line treatment for ES-SCLC.

## Methods

### Study design and patients

This research is a multicenter, non-intervention, retrospective real-world study. ES-SCLC patients receiving anlotinib combined with platinum-etoposide chemotherapy as the first-line treatment in the First Affiliated Hospital of Xi’an Jiaotong University, Xijing Hospital of Air Force Military Medical University, Xianyang Central Hospital, Shaanxi Nuclear Industry 215 Hospital, Hanzhong Central Hospital, and Baoji Traditional Chinese Medicine Hospital were eligible for retrospective analysis between December 1, 2018, and July 31, 2021. These tertiary hospitals are located in Shaanxi, China. The characteristics of patients were collected, including age, sex, smoking status, ECOG PS, age-adjusted Charlson comorbidity index (aCCI), TNM stage, number and location of metastases, anlotinib initial dose, imaging and laboratory examination, and adverse reaction.

### Inclusion and exclusion criteria

The inclusion criteria for patients were as follows: (1) age ≥18 years; (2) patients with ES-SCLC diagnosed by pathology have measurable lesions according to the Response Evaluation Criteria in Solid Tumors (RECIST) version 1.1 standard; (3) receiving anlotinib combined with platinum-etoposide chemotherapy as the first-line treatment; (4) ECOG PS ≤2; (5) without surgery. The exclusion criteria for patients were as follows: (1) severe lack of clinical records or loss of follow-up; (2) imaging efficacy evaluation cannot be performed; (3) patients with active bleeding or serious systemic diseases.

### Therapeutic methods

Each patient was treated with 2 to 8 21-day cycles of anlotinib (12mg/10mg, day 1 to 14 of each cycle), etoposide (100mg/m^2^ of body surface area, day 1 to 3 of each cycle), and carboplatin (area under the curve of 5mg/mL/min, day 1 of each cycle) or cisplatin (25mg/m^2^ of body surface area, day 1 to 3 of each cycle), followed by anlotinib maintenance every 3 weeks. The actual dosage was adjusted by qualified physicians according to patients’ situation. Treatment was continued until disease progression, death, or unacceptable toxicity.

### Efficacy and safety evaluation

According to the RECIST version 1.1 standard, two qualified physicians independently evaluated the efficacy through computed tomography (CT) or magnetic resonance imaging (MRI). The responses were classified as complete response (CR), partial response (PR), stable disease (SD), or progressive disease (PD). When there was disagreement on the assessment, a third physician was requested to reevaluate. Follow-up data were collected up to October 31, 2021. PFS was defined as the time from the start of treatment until tumor progression or death from any cause before disease progression or last follow-up. OS was defined as the time from the treatment initiation to death or last follow-up. Respectively, ORR or DCR was calculated as the addition of CRs plus PRs or CRs plus PRs plus SDs. The adverse reactions were graded according to the Common Terminology Criteria for Adverse Events (CTCAE) version 4.0. The primary endpoint of this study was PFS, and secondary endpoints included OS, ORR, DCR, and adverse reactions.

### Statistical analysis

Patients’ baseline characteristics were summarized as proportions for categorical variables and medians (range) for continuous variables as appropriate. The median PFS, OS, and 95% confidence interval (CI) were estimated using the Kaplan–Meier method. Cox proportional hazards regression was used for the univariable and multivariable analyses and to calculate the hazard ratios (HR) with 95% CIs. All statistical analyses in this study were performed using SPSS version 18.0 for Windows 64.0 and GraphPad Prism version 6.0. A two-tailed *P*-value <0.05 was considered statistically different.

## Results

### Baseline clinical characteristics of patients

In total, 58 patients were included in the present study. Among them, 11 (19.0%) patients were from the First Affiliated Hospital of Xi’an Jiaotong University, 12 (20.7%) patients were from Xijing Hospital of Air Force Military Medical University, 21 (36.2%) patients were from Xianyang Central Hospital, 7 (12.1%) patients were from Shaanxi Nuclear Industry 215 Hospital, 4 (6.9%) patients were from Hanzhong Central Hospital, and 3(5.1%) patients were from Baoji Traditional Chinese Medicine Hospital. The median follow-up duration was 7.9 months. Details of the patients’ baseline clinical characteristics were shown in **Table 1**. The median age of the patients was 59 years (range, 36 to 81 years). A total of 47 patients were male (81.0%). Former smokers and non-smokers were noted in 41 (70.7%) and 17 (29.3%) patients. ECOG PS 0-1 were observed in 38 (65.5%) patients. Forty-three (74.1%) patients were initially diagnosed in the TNM IV stage. Among them, 24 (41.4%) patients received thoracic radiotherapy during the treatment. In addition, patients with post-medication hypertension and hand-foot syndrome were observed in 24 (41.4%) cases and 14 (24.1%) cases, respectively.

**Table 1:** Baseline clinical characteristics of patients.

Characteristics	N (%)
Age (years)	
Median (range)	59 (36-81)
<65	41 (70.7)
≥65	17 (29.3)
Sex	
Male	47 (81.0)
Female	11 (19.0)
Smoking status	
Ever	41 (70.7)
Never	17 (29.3)
ECOG PS	
0-1	38 (65.5)
2	20 (34.5)
aCCI	
<8	26 (44.8)
≥8	32 (55.2)
TNM stage	
III	15 (25.9)
IV	43 (74.1)
T stage	
T1-2	23 (39.7)
T3-4	35 (60.3)
N stage	
N0-2	9 (15.5)
N3	49 (84.5)
Number of metastatic sites	
<2	33 (56.9)
≥2	25 (43.1)
Brain metastases	
Yes	5 (8.6)
No	53 (91.4)
Hepatic metastases	
Yes	16 (27.6)
No	42 (72.4)
Osseous metastases	
Yes	14 (24.1)
No	44 (75.9)
Pleural metastases/pleural effusion	
Yes	20 (34.5)
No	38 (65.5)
Lung metastases	
Yes	19 (32.8)
No	39 (67.2)
Baseline NSE	
≤20ng/ml	19 (32.8)
>20ng/ml	39 (67.2)
Anlotinib initial dose	
10mg	4 (6.9)
12mg	54 (93.1)
Plus thoracic radiotherapy	
Yes	24 (41.4)
No	34 (58.6)
Post-medication hypertension	
Yes	24 (41.4)
No	34 (58.6)
Post-medication hand-foot syndrome	
Yes	14 (24.1)
No	44 (75.9)

ECOG PS, Eastern Cooperative Oncology Group Performance Status; aCCI, age-adjusted Charlson comorbidity index; NSE, neuron specific enolase.

### Clinical efficacy

**Figure 1:**
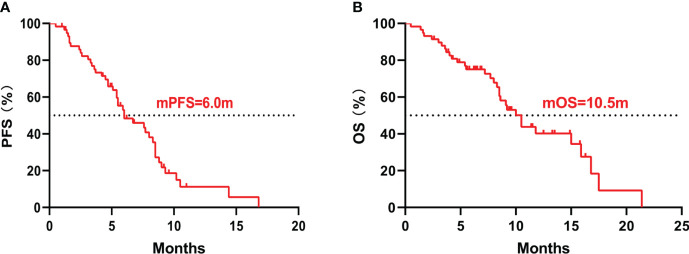
Kaplan–Meier curves of all patients. **(A)** The Kaplan–Meier curve of PFS; **(B)** The Kaplan–Meier curve of OS. PFS, progression-free survival; OS, overall survival.

**Figure 2:**
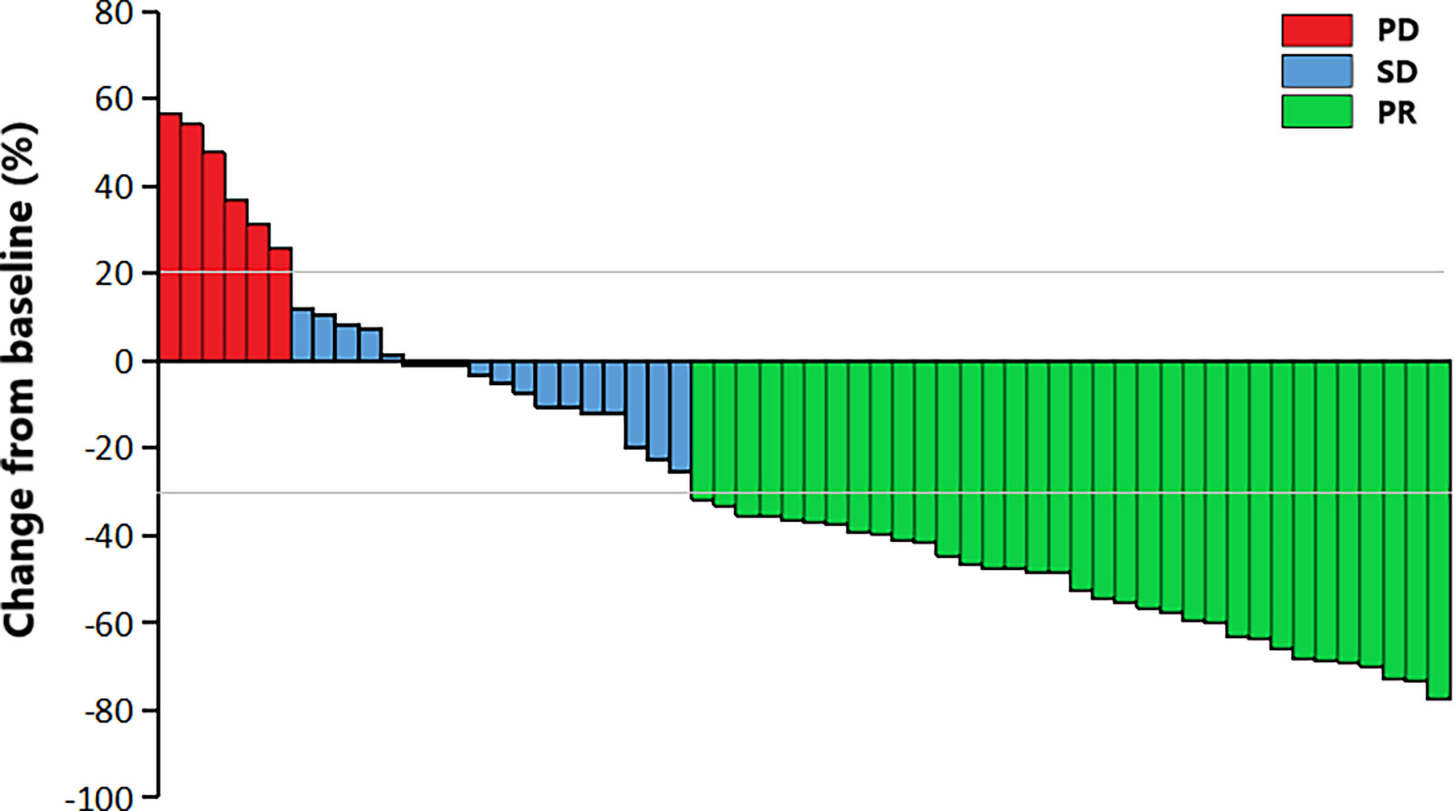
The waterfall plot of tumor best response compared with baseline measurable lesions. PR, partial response; SD, stable disease; PD, progressive disease.

The median PFS was 6.0 months (95%CI 3.5-8.5), and the median OS was 10.5 months (95%CI 8.7-12.3) (**Figure 1A, B**). The 6-month PFS rate was 47.9%, the 6-month OS rate was 72.5%, and the 1-year OS rate was 28.9%. Among them, 34 (58.6%) patients achieved PR, 18 (31.0%) patients achieved SD, and 6 (10.4%) patients achieved PD. Respectively, the ORR and DCR were 58.6% and 89.6%. The waterfall plot of tumor best response compared with measurable baseline lesions was shown in **Figure 2**. A 52-year-old female patient without metastasis reached the longest PFS of 16.8 months.

**Figure 3:**
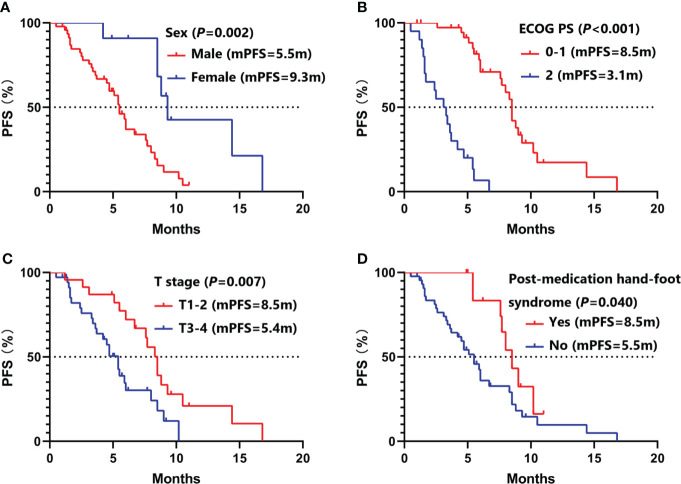
Kaplan–Meier curves of PFS in multivariate Cox regression analysis. **(A)** stratified by sex; **(B)** stratified by ECOG PS; **(C)** stratified by T stage; **(D)** stratified by post-medication hand-foot syndrome. PFS, progression-free survival; ECOG PS, Eastern Cooperative Oncology Group Performance Status.

**Figure 4:**
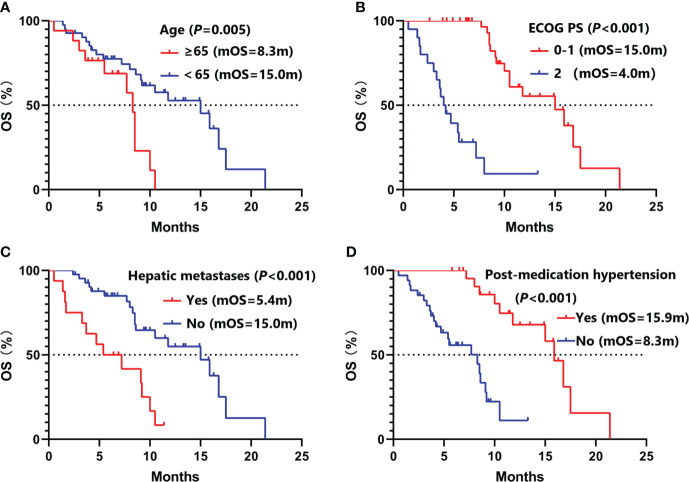
Kaplan–Meier curves of OS in multivariate Cox regression analysis. **(A)** stratified by age; **(B)** stratified by ECOG PS; **(C)** stratified by hepatic metastases; **(D)** stratified by post-medication hypertension. OS, overall survival; ECOG PS, Eastern Cooperative Oncology Group Performance Status.

Univariate analysis (**Table 2**) showed that female (9.3 vs. 5.5 months, *P* =0.002), ECOG PS 0-1 (8.5 vs. 3.1 months, *P <*0.001), aCCI <8 (8.0 vs. 5.5 months, *P* =0.044), T1-2 (8.5 vs. 5.4 months, *P* =0.007), no hepatic metastases (8.0 vs. 4.7 months, *P* =0.010), baseline neuron specific enolase (NSE) ≤20ng/ml (8.5 vs. 5.4 months, *P* =0.006), plus thoracic radiotherapy (8.3 vs. 4.2 months, *P* =0.002), post-medication hypertension (8.5 vs. 5.4months, *P* =0.008), and post-medication hand-foot syndrome (8.5 vs. 5.5 months, *P* =0.040) might have longer PFS benefits. Age <65 (15.0 vs. 8.3 months, *P* =0.005), female (16.8 *vs.* 9.1 months, *P* =0.009), never smoking (16.8 *vs.* 9.1 months, *P* =0.024), ECOG PS 0-1 (15.0 *vs.* 4.0 months, *P <*0.001), aCCI < 8 (15.9 vs. 8.5 months, *P* =0.013), N0-2 (17.5 vs. 9.2 months, *P* =0.043), no hepatic metastases (15.0 *vs.* 5.4 months, *P <*0.001), plus thoracic radiotherapy (16.8 *vs.* 7.7 months, *P <*0.001), and post-medication hypertension (15.9 *vs.* 8.3 months, *P <*0.001) might have longer OS benefits. Factors with *P* < 0.050 in univariate analyses were included in multivariate Cox regression analysis. Multivariate analysis revealed that sex (male *vs.* female: HR=6.05, 95%CI 1.74-20.98, *P* =0.005), ECOG PS (2 *vs.* 0-1: HR=8.34, 95%CI 2.54-27.39, *P <*0.001), T stage (T3-4 *vs.* T1-2: HR=3.82, 95%CI 1.59-9.18, *P* =0.003), and post-medication hand-foot syndrome (yes *vs.* no: HR=0.23, 95%CI 0.07-0.72, *P* =0.012) were the independent predictors of PFS (**Table 3**). Age (≥65 *vs.* <65: HR=4.87, 95%CI 1.71-13.82, *P* =0.003), ECOG PS (2 *vs.* 0-1: HR=11.26, 95%CI 2.49-50.84, *P* =0.002), hepatic metastases (yes *vs.* no: HR=3.83, 95%CI 1.41-10.41, *P* =0.008), and post-medication hypertension (yes *vs.* no: HR=0.18, 95%CI 0.05-0.58, *P* =0.005) were the independent predictors of OS (**Table 4**). The Kaplan–Meier curves of PFS and OS in multivariate Cox regression analysis were presented in **Figures 3, 4**.

**Table 2:** Univariate analysis of factors associated with PFS and OS.

Factors	mPFS (months)	95% CI	*P*-value	mOS (months)	95% CI	*P*-value
Age (years)			0.262			**0.005**
<65	6.0	3.3-8.7		15.0	9.8-20.2	
≥65	5.9	2.7-9.1		8.3	7.6-9.0	
Sex			**0.002**			**0.009**
Male	5.5	4.6-6.4		9.1	8.0-10.2	
Female	9.3	8.1-10.5		16.8	10.5-23.1	
Smoking status			0.084			**0.024**
Ever	6.0	5.3-6.7		9.1	8.0-10.2	
Never	8.5	4.0-13.0		16.8	5.6-28.0	
ECOG PS			**< 0.001**			**< 0.001**
0-1	8.5	7.9-9.1		15.0	9.7-20.3	
2	3.1	1.3-4.9		4.0	2.9-5.1	
aCCI			**0.044**			**0.013**
<8	8.0	7.1-8.9		15.9	9.1-22.7	
≥8	5.5	3.2-7.8		8.5	6.5-10.5	
TNM stage			0.217			0.663
III	8.3	7.8-8.8		8.5	5.1-11.9	
IV	5.4	4.1-6.7		10.5	8.7-12.3	
T stage			**0.007**			0.631
T1-2	8.5	7.4-9.6		9.2	6.6-11.8	
T3-4	5.4	4.3-6.5		11.8	9.1-14.5	
N stage			0.129			**0.043**
N0-2	9.3	4.4-14.2		17.5	8.6-26.4	
N3	6.0	5.3-6.7		9.2	7.5-10.9	
Number of metastatic sites			0.114			0.226
<2	8.3	7.6-9.0		11.8	6.2-17.4	
≥2	5.4	4.9-5.9		9.2	7.7-10.7	
Brain metastases			0.851			0.506
Yes	6.7	3.3-10.1		8.6	1.0-16.2	
No	6.0	3.0-9.0		10.5	8.8-12.2	
Hepatic metastases			**0.010**			**< 0.001**
Yes	4.7	3.2-6.2		5.4	1.2-9.6	
No	8.0	6.1-9.9		15.0	9.8-20.2	
Osseous metastases			0.238			0.287
Yes	5.4	4.9-5.9		9.1	4.4-13.8	
No	7.7	5.1-10.3		10.5	6.6-14.4	
Pleural metastases/pleural effusion			0.132			0.700
Yes	5.4	4.0-6.8		9.2	–	
No	8.0	5.5-10.5		10.5	7.9-13.1	
Lung metastases			0.849			0.912
Yes	5.1	3.0-7.2		10.5	3.6-17.4	
No	6.7	4.1-9.3		9.2	7.0-11.4	
Baseline NSE			**0.006**			0.051
≤20ng/ml	8.5	7.8-9.2		21.4	–	
>20ng/ml	5.4	4.6-6.2		9.2	7.1-11.3	
Anlotinib initialdose			0.970			0.534
10mg	2.6	0.0-6.9		8.6	1.9-15.3	
12mg	6.7	4.3-9.1		10.5	7.5-13.5	
Plus thoracic radiotherapy			**0.002**			**<0.001**
Yes	8.3	6.6-10.0		16.8	14.7-18.9	
No	4.2	1.9-6.5		7.7	3.9-11.5	
Post-medication hypertension			**0.008**			**<0.001**
Yes	8.5	6.1-10.9		15.9	14.0-17.8	
No	5.4	3.4-7.4		8.3	3.3-13.3	
Post-medication hand-foot syndrome			**0.040**			0.115
Yes	8.5	7.4-9.6		15.9	9.9-21.9	
No	5.5	4.2-6.8		9.2	7.1-11.3	

PFS, progression-free survival; OS, overall survival; CI, confidence interval; ECOG PS, Eastern Cooperative Oncology Group Performance Status; aCCI, age-adjusted Charlson comorbidity index; NSE, neuron specific enolase.

Bold value represents P-value < 0.05.

**Table 3:** Multivariate Cox regression analysis of factors associated with PFS.

Factors	HR	95%CI	*P*-value
Sex (Male *vs.* Female)	6.05	1.74-20.98	**0.005**
ECOG PS (2 *vs.* 0-1)	8.34	2.54-27.39	**<0.001**
aCCI (≥8 *vs.* <8)	1.51	0.74-3.06	0.257
T stage (T3-4 *vs.* T1-2)	3.82	1.59-9.18	**0.003**
Hepatic metastases (Yes *vs.* No)	0.67	0.30-1.49	0.323
Baseline NSE (>20ng/ml *vs.* ≤20ng/ml)	1.39	0.47-4.06	0.551
Plus thoracic radiotherapy (Yes *vs.* No)	0.98	0.30-3.23	0.979
Post-medication hypertension (Yes *vs.* No)	0.72	0.31-1.68	0.450
Post-medication hand-foot syndrome (Yes *vs.* No)	0.23	0.07-0.72	**0.012**

PFS, progression-free survival; HR, Hazard ratio; CI, Confidence inter; ECOG PS, Eastern Cooperative Oncology Group Performance Status; aCCI, age-adjusted Charlson comorbidity index; NSE, neuron specific enolase.

Bold value represents P-value < 0.05.

**Table 4:** Multivariate Cox regression analysis of factors associated with OS.

Factors	HR	95%CI	*P*-value
Age (≥65 *vs.* <65)	4.87	1.71-13.82	**0.003**
Sex (Male *vs.* Female)	0.88	0.13-5.83	0.891
Smoking status (Ever *vs.* Never)	2.52	0.51-12.49	0.258
ECOG PS (2 *vs.* 0-1)	11.26	2.49-50.84	**0.002**
aCCI (≥8 *vs.* <8)	1.89	0.69-5.13	0.213
N stage (N3 *vs.* N0-2)	0.90	0.18-4.57	0.899
Hepatic metastases (Yes *vs.* No)	3.83	1.41-10.41	**0.008**
Plus thoracic radiotherapy (Yes *vs.* No)	0.73	0.17-3.04	0.662
Post-medication hypertension (Yes *vs.* No)	0.18	0.05-0.58	**0.005**

OS, overall survival; HR, Hazard ratio; CI, Confidence inter; ECOG PS, Eastern Cooperative Oncology Group Performance Status; aCCI, age-adjusted Charlson comorbidity index.

Bold value represents P-value < 0.05.

Patients with ECOG PS ≥2 are often excluded in clinical trials. But 20 (34.5%) patients with ECOG PS 2 were included in this study. In univariate and multivariate Cox regression analysis, we found that ECOG PS was the independent predictors of PFS and OS. Similar with other clinical trials, patients with ECOG PS 0-1 had longer PFS (8.5 *vs.* 3.1 months, *P <*0.001) and OS (15.0 *vs.* 4.0 months, *P <*0.001) than patients with ECOG PS 2 (**Figure 3B**, **Figure 4B**). Of all 38 patients with ECOG PS 0-1, the 6-month PFS rate was 75.9%, the 6-month OS rate was 100.0%, and the 1-year OS rate was 62.5%. Among them, 28 (73.7%) patients achieved PR, 9 (23.7%) patients achieved SD, and 1 (2.6%) patient achieved PD. Respectively, the ORR and DCR were 73.7% and 97.4% (**Table 5**).

**Table 5:** Efficacy in patients with different ECOG PS.

	Overall (n=58)	ECOG PS 0-1 (n=38)	ECOG PS 2 (n=20)
PR, n(%)	34 (58.6)	28 (73.7)	6 (30.0)
SD, n(%)	18 (31.0)	9 (23.7)	9 (45.0)
PD, n(%)	6 (10.4)	1 (2.6)	5 (25.0)
ORR, %	58.6	73.7	30.0
DCR, %	89.6	97.4	75.0
mPFS (months)	6.0	8.5	3.1
mOS (months)	10.5	15.0	4.0
6-month PFS, %	47.9	75.9	5.3
6-month OS, %	72.5	100.0	22.2
1-year OS, %	28.9	62.5	5.9

ECOG PS, Eastern Cooperative Oncology Group Performance Status; PR, partial response; SD, stable disease; PD, progressive disease; ORR, objective response rate; DCR, disease control rate; PFS, progression-free survival; OS, overall survival.

### Safety

All of the 58 patients were available for safety profile. The incidence of treatment-related adverse reactions was 70.7% (41/58), and the incidence of grade 3 and above adverse reactions was 24.1% (14/58) among the participants. Dose reductions due to adverse reactions were required for 16 (27.6%) patients, and 7 (12.1%) patients discontinued the treatment. There were no treatment-related deaths in this research. As shown in **Table 6**, the most common adverse reaction was myelosuppression (44.8%), followed by hypertension (41.4%), fatigue (34.5%), gastrointestinal reaction (32.7%), and hand-foot syndrome (24.1%). Notably, most of the adverse reactions were grade 1-2.

**Table 6:** Summary of adverse reactions.

Toxicity	All grades (%)	Grade 1-2 (%)	Grade ≥3 (%)
Myelosuppression	26 (44.8)	17 (29.3)	9 (15.5)
Hypertension	24 (41.4)	21 (36.2)	3 (5.2)
Fatigue	20 (34.5)	20 (34.5)	0 (0.0)
Gastrointestinal reaction	19 (32.7)	17 (29.3)	2 (3.4)
Hand-foot syndrome	14 (24.1)	13 (22.4)	1 (1.7)
Hyperlipemia	11 (19.0)	11 (19.0)	0 (0.0)
Hemorrhage	9 (15.5)	6 (10.3)	3 (5.2)
Transaminase elevation	6 (10.3)	5 (8.6)	1 (1.7)
Hyponatremia	6 (10.3)	6 (10.3)	0 (0.0)
Hyperbilirubinemia	6 (10.3)	4 (6.9)	2 (3.4)
Hypophosphatemia	5 (8.6)	4 (6.9)	1 (1.7)
Mucositis oral	5 (8.6)	4 (6.9)	1 (1.7)
Rash	4 (6.9)	4 (6.9)	0 (0.0)
Thyroid dysfunction	3 (5.2)	3 (5.2)	0 (0.0)
Hypokalemia	3 (5.2)	3 (5.2)	0 (0.0)
Proteinuria	3 (5.2)	3 (5.2)	0 (0.0)
Hoarseness	1 (1.7)	1 (1.7)	0 (0.0)
Arthralgia	1 (1.7)	1 (1.7)	0 (0.0)

## Discussion

SCLC has an abnormally high proliferation rate, a strong tendency for early metastasis, and a bleak prognosis (4). As the first-line standard treatment for ES-SCLC in the past 40 years, the PFS of platinum-etoposide chemotherapy is about 5 months, and the median OS is about 10 months (8). Based on the IMpower133 and CASPIAN clinical trials, PD-L1 plus platinum-etoposide chemotherapy has become the new first-line standard therapy in recent years. Although, it only brought the OS benefit for 2 to 3 months (9, 10). Angiogenesis serves a pivotal role in tumor occurrence, invasion, and metastasis (21). However, the efficacy of antiangiogenic therapy in SCLC is limited, such as bevacizumab, sorafenib, sunitinib and so on, except for anlotinib (15, 22–24). In China, anlotinib has been approved by CFDA as the third-line and above treatment for SCLC based on the ALTER 1202 study (17). Several small sample size single arm phase II clinical trials of anlotinib combined with platinum-etoposide chemotherapy as the first-line treatment for ES-SCLC are being carried out in China and the preliminary results have shown the favorable clinical efficacy (18–20).

Antiangiogenic therapy can improve drug delivery efficiency by opening the vascular normalization window, thus exerting a synergistic effect when combined with other regimens (25). In addition, the non-overlapping toxicity spectrum and excellent tolerance of anlotinib allow it to be used in combination with other drugs. In a clinical trial conducted by Kong T et al., 20 ES-SCLC patients received anlotinib plus platinum-etoposide chemotherapy as the first-line therapy, the median PFS was 10.0 months, and the median OS was 15.0 months (18). Similarly, in Deng P’s study, the median PFS and OS were 9.4 and 13.9 months, respectively (20). Supported by these encouraging preliminary results, phase III clinical trials have already begun in China. In this real-world study, the median PFS was 6.0 months, the median OS was 10.5 months, the ORR was 58.6%, and the DCR was 89.6%. Our results are similar to the efficacy of traditional platinum-etoposide chemotherapy. But 20 (34.5%) patients with ECOG PS 2 were included in this study. In contrast, there were no patients with ECOG PS >1 in these clinical trials. The median PFS and OS of patients with ECOG PS 0-1 in this study were 8.5 and 15.0 months, respectively. The 6-month PFS rate was 75.9%, the 6-month OS rate was 100.0%, and the 1-year OS rate was 62.5%. Respectively, the ORR and DCR were 73.7% and 97.4%. Multivariate Cox regression analysis showed that ECOG PS was the independent influencing factor of PFS and OS. This result showed better efficacy compared with traditional chemotherapy and PD-L1 plus chemotherapy, and the OS was similar to the clinical studies of anlotinib plus platinum-etoposide chemotherapy. Since there is no control group in our study, the efficacy of combination therapy still requires further verification by prospective studies with larger sample size.

ES-SCLC patients first receive chemotherapy to control the spread of metastasis. Subsequently, chest radiotherapy is recommended to control local lesions for patients who achieve CR or PR after chemotherapy (26). Some studies found that antiangiogenic therapy can increase the local oxygen partial pressure and oxygen content of tumor tissue, inhibit the angiogenesis induced by radiotherapy, and play the role of radiotherapy sensitization (27). In our study, patients combined with thoracic radiotherapy had more extended PFS (8.3 *vs.* 4.2 months, *P* =0.002) and OS (16.8 *vs.* 7.7 months, *P <*0.001) benefits in univariate analysis. However, there were no statistical differences in multivariate analysis.

Hypertension and hand-foot syndrome are the most common adverse reactions of anlotinib. Interestingly, more extended PFS benefits were observed in ES-SCLC patients with post-medication hypertension or hand-foot syndrome in Song PF’s study (28). In this research, patients with post-medication hypertension (8.5 vs. 5.4 months, *P* =0.008) and hand-foot syndrome (8.5 vs. 5.5 months, *P* =0.040) had longer PFS benefits in univariate analysis. Additionally, we also found that patients with post-medication hypertension (15.9 vs. 8.3 months, *P <*0.001) had longer OS benefits. Multivariate analysis showed that post-medication hand-foot syndrome (yes vs. no: HR=0.23, 95%CI 0.07-0.72, *P* =0.012) was the independent predictor of PFS, and post-medication hypertension (yes vs. no: HR=0.18, 95%CI 0.05-0.58, *P* =0.005) was the independent predictor of OS. Hypertension might be attributed to the mechanism that inhibition of VEGFR in vascular endothelial cells decreased the production of nitricoxide and prostacyclins, thus leading to increased blood pressure (29). Hand-foot syndrome might be induced by decreased reconstruction of skin after restriction of vessels (30). Therefore, hypertension or hand-foot syndrome induced by anlotinib could partly reflect the inherent host biology that caused differences in VEGF/VEGFR blockade (31).

Furthermore, we observed that sex (male vs. female: HR=6.05, 95%CI 1.74-20.98, *P* =0.005) and T stage (T3-4 vs. T1-2: HR=3.82, 95%CI 1.59-9.18, *P* =0.003) were the independent influencing factors of PFS. Age (≥65 vs. <65: HR=4.87, 95%CI 1.71-13.82, *P* =0.003) and hepatic metastases (yes vs. no: HR=3.83, 95%CI 1.41-10.41, *P* =0.008) were associated with OS in multivariate Cox regression analysis. However, only 11 (19.0%) female patients were included in our study, which might influence the result.

In this research, the toxicity of anlotinib plus platinum-etoposide chemotherapy was generally well tolerated. The grade 3 and above adverse reactions were manageable with dose reduction or drug discontinuation. Similar to previous research, myelosuppression was the most frequent adverse reaction (18–20). As the most common adverse reactions of anlotinib, the incidence of hypertension and hand-foot syndrome were 41.1% and 24.1%, respectively. There were no new anlotinib-related adverse reactions observed in this study, and the toxic profile was similar to other studies of anlotinib in SCLC (17). The incidence of adverse reactions in this research might be lower than actual data in the real world because of the bias of the retrospective study.

This study provided real-world data of anlotinib combined with platinum-etoposide chemotherapy as the first-line treatment for ES-SCLC at the first time. Despite the advantages of this work, there are several inevitable shortcomings in our study. First, as a real-world study, the Chinese undiversified population and small sample size might affect the universality of the results. Thus, well-designed large-scale prospective studies are urgently needed in the future to provide more profound insights into this field. Second, due to the retrospective design of this study, selection bias and information bias could not be avoided. For instance, the majority of patients included in this study are male, which may affect the representation of the study population. Besides, although we identified that post-medication hypertension and foot-hand syndrome may correlated with favorable prognosis after treatment, the sample size of these patients is small and some adverse effects are not well recorded. This further emphasizes the importance of conducting relevant studies in the future. Last but not least, since the dosage was determined by different physicians according to the actual situation of patients, and this may affect the efficacy.

## Conclusion

To sum up, our study revealed that anlotinib combined with platinum-etoposide chemotherapy as the first-line treatment for ES-SCLC appears to be effective and well-tolerated in the real-world setting, especially in patients with ECOG PS 0-1. Patients with post-medication hypertension and hand-foot syndrome may confer superior survival benefits. However, well-designed large-scale prospective studies are urgently needed in the future to verify our findings.

## Data availability statement

The original contributions presented in the study are included in the article/supplementary material. Further inquiries can be directed to the corresponding author.

## Ethics statement

The studies involving human participants were reviewed and approved by Ethics Committee of the First Affiliated Hospital of Xi’an Jiaotong University. Written informed consent for participation was not required for this study in accordance with the national legislation and the institutional requirements.

## Author contributions

YY contributed to the concept and design of the research; H-RZ established the database; H-RZ, A-MJ, and HG conducted statistical analysis; H-RZ and A-MJ wrote the first draft of the manuscript; HG, NL, X-QZ, XF, Z-PR, TT, XL and YY reviewed and edited the manuscript. All authors participated in the revision of the manuscript, read and approved the submitted version.

## Funding

This study was funded by International Cooperation Project in Science and Technology of Shaanxi Province (No. 2019KW-074), Nation Natural Science Funding of China (No. 82002437), and Shaanxi Province Technology Innovation Team (No. 2021TD-44).

## Acknowledgments

We would like to thank Xijing Hospital of Air Force Military Medical University, Xianyang Central Hospital, Shaanxi Nuclear Industry 215 Hospital, Hanzhong Central Hospital and Baoji Traditional Chinese Medicine Hospital for providing the clinical data.

## Conflict of interest

The authors declare that the research was conducted in the absence of any commercial or financial relationships that could be construed as a potential conflict of interest.

## Publisher’s note

All claims expressed in this article are solely those of the authors and do not necessarily represent those of their affiliated organizations, or those of the publisher, the editors and the reviewers. Any product that may be evaluated in this article, or claim that may be made by its manufacturer, is not guaranteed or endorsed by the publisher.
